# Urological Cancers in French Overseas Territories: A Population-Based Cancer Registry Pooled Analysis in Martinique, Guadeloupe and French Guiana (2007–2014)

**DOI:** 10.1007/s44197-022-00033-9

**Published:** 2022-01-18

**Authors:** Jacqueline Deloumeaux, Bernard Bhakkan-Mambir, Laure Desroziers, Juliette Plenet, Jessica Peruvien, Édouard Chatignoux, Sophie Belliardo, Jonathan Macni, Stephen Ulric-Gervaise, Jacqueline Véronique-Baudin, Clarisse Joachim

**Affiliations:** 1 Registre Général des Cancers de Guadeloupe, Centre Hospitalier Universitaire de Guadeloupe, Guadeloupe F.W. I., Route de Chauvel, 97159 Pointe-à-Pitre Cedex, France; 2 Registre Général des Cancers de la Guyane, Guyane, France; 3 French National Public Health Agency, 12 rue du Val d’Osne, 94410 Saint Maurice, France; 4 CHU de Martinique, Pôle de Cancérologie Hématologie Urologie, UF 1441 Registre Général des Cancers de la Martinique, Fort de France, 97200 Martinique; 5 Hôpital Albert Clarac, Avenue Pasteur, CS 90632, 97200 Fort de France, Martinique

**Keywords:** Prostate, Urology, Cancer registry, Incidence, Caribbean

## Abstract

**Purpose:**

Prostate cancer is the most common cancer in the Caribbean. We present world-standardized incidence (WSI) and mortality (WSM) rates for urological cancers for French overseas territories.

**Materials and Methods:**

Standardized incidence ratio (SIR) and standardized mortality ratio (SMR) were calculated for 2008–2014, 2007–2014 and 2010–2014 in Guadeloupe, Martinique and French Guiana.

**Results:**

For prostate cancer, in Guadeloupe and Martinique, the WSI rates are among the highest in the world (173.0 and 164.5 per 100,000 person-years) and 94.4 in French Guiana. Mortality remains more than twice that observed in mainland France, at 23.0 in Guadeloupe and Martinique, and 16.9 in French Guiana.

For bladder cancer, WSI rates were 5.9, 4.9 and 4.1 in men, and 1.9, 1.4 and 1.3 in women, in French Guiana, Guadeloupe and Martinique. WSM rates from bladder varied from 1.5 in French Guiana to 1.8 in Guadeloupe and 2.0 in Martinique in men. In women, it ranges from 0.2 in French Guiana to 0.5 in Guadeloupe and 1.1 in Martinique. Regarding kidney, WSI rates in men are 4.3 in Martinique, 5.2 in Guadeloupe and 6.1 in French Guiana, and 2.3, 2.5 and 3.4, respectively, in women. Mortality rates in men were 1.7 in Guadeloupe, 1.4 in Martinique, and 1.5 in French Guiana, while in women, rates were 0.8 in Guadeloupe and Martinique and 0.6 in French Guiana. All these rates are lower than in mainland France.

**Conclusions:**

Identifying the profile of patients with urological cancers is key to understanding the needs of patients in these regions.

## Introduction

Incidence and mortality data show that prostate cancer is a major public health problem in the Caribbean region, where it is the most common cancer in men [1]. The French overseas territories are in need of reliable and regularly updated epidemiological data to help inform public health policies to fight cancer [2]. As a means of generating reliable epidemiological data, population-based cancer registries (PBCRs) make a key contribution to producing public health indicators, notably incidence, mortality and survival data [3, 4]. These indicators are useful for patients, researchers and health authorities and decision-makers, and can be made available via scientific publications, or published on the websites of public institutions such as the French public health authority (Santé Publique France) or the national cancer institute (Institut National du Cancer, INCa) [5].

Available data reveal a very particular epidemiological profile in the French overseas territories as compared with mainland France, with excess incidence and mortality observed for prostate cancer. However, observational studies and analyses of net survival at 5 years have shown that prostate cancer has a generally good prognosis [6–10]. Furthermore, in the framework of public health campaigns relating to the use of chlordecone, various studies have underlined the need for surveillance of population health due to persistent presence of organochlorine pesticides [11, 12].

Other urological cancers, such as kidney and bladder cancer, have lower incidence and mortality rates, but may also warrant surveillance via dedicated networks. Smoking is the main risk factor for bladder cancer in Europe [13], while tobacco consumption is lower in the French west-indies [14].

Occupational exposures (aromatic amines, polycyclic aromatic hydrocarbons, etc.) are the second major risk factor. A genetic predisposition may also be involved.

Conversely, the consumption of fruit and vegetables, and regular physical exercise are thought to have a protective effect [15, 16].

Previous works in the French overseas territories over the last decade in the framework of the French anti-cancer plan for the period 2014–2019 [17] have underlined the pressing need to produce regular indicators of incidence and mortality for the three French regions of the Caribbean, namely Martinique, Guadeloupe and French Guiana. To meet this need, our study presents world-standardized cancer incidence and mortality rates for urological cancers in Guadeloupe, French Guiana and Martinique for the period 2007–2014.

## Materials and Methods

The data are from the general cancer registries of Martinique, Guadeloupe and French Guiana. In these registries, all variables are recorded, verified and analyzed according to national and international standard procedures, guaranteeing the quality of the data collected. All three registries have been audited and approved by the national committee for the evaluation of registries, for their high-quality contribution to public health data, the quality of the data available, and the extent of the research developed within the registries.

The periods 2008–2014, 2007–2014 and 2010–2014 were analyzed, respectively, for Guadeloupe, Martinique and French Guiana. Incidence data for mainland France were produced using a calibration model [18]. Mortality data were obtained from the Centre for Epidemiology of the medical causes of death (CepiDC). Mortality data for 2012 were not included for Martinique; thus, the mortality rates for 2007–2014 do not include this year for Martinique.

We calculated the standardized incidence and mortality rates. The standardized incidence ratio (SIR) or standardized mortality ratio (SMR) were calculated from 1960 as the standard population [10]. The SIR (respectively, SMR), in a given geographical area (department or region), is the ratio between the number of estimated incident cases (resp. deaths) and the expected number of cases (resp. deaths) if the age-specific incidence (resp. mortality) rates in this geographical area were identical to those in mainland France.

## Results

### Prostate

In the French Overseas territories, prostate cancer ranks first among the male cancers, as in mainland France. With more than 500 new cases diagnosed per year in Guadeloupe and Martinique, respectively (Table 1), prostate cancer represents 35% of all cancers, and more than 55% of male cancers. In French Guiana, prostate cancer is diagnosed in an average of 78 men per year (Table 1) and represents 17% of all cancers, and 32% of male cancers.

**Table 1:** Annual number of new cases and deaths for prostate, bladder and kidney cancers, standardized incidence and mortality rates, standardized incidence and mortality ratios, with 95% confidence intervals

	Incidence						Mortality					
	New cases^a^		WSR^b^		SIR^c^		Deaths		WSR^b^		SMR^c^	
Prostate												
Guadeloupe	542	[525; 560]	173.0	[167.4; 178.9]	1.91	[1.85; 1.97]	99	[92; 106]	23.1	[21.5; 25.0]	2.28	[2.13; 2.45]
Martinique	530	[514; 546]	164.5	[159.4; 169.8]	1.79	[1.73; 1.84]	109	[102; 117]	23.2	[21.5; 25.2]	2.37	[2.20; 2.54]
French Guiana	78	[70; 86]	94.4	[85.0; 104.6]	1.04	[0.94; 1.15]	12	[10; 15]	16.9	[13.7; 20.7]	1.70	[1.38; 2.07]
Mainland France	51 024	[50 387; 51 672]	88.8	[87.7; 90.9]			8787	[8723; 8853]	10.0	[9.9; 10.1]		
Bladder in men												
Guadeloupe	16	[13; 19]	4.9	[4.0; 6.1]	0.32	[0.26; 0.38]	6	[5; 8]	1.8	[1.3; 2.5]	0.33	[0.25; 0.44]
Martinique	15	[12; 18]	4.1	[3.3; 5.1]	0.28	[0.23; 0.34]	9	[7; 11]	2.0	[1.5; 2.8]	0.43	[0.33; 0.55]
French Guiana	5	[3; 8]	5.9	[3.8; 8.8]	0.44	[0.28; 0.64]	1	[1; 2]	1.5	[0.6; 2.9]	0.29	[0.13; 0.56]
Mainland France	9441	[9165; 9728]	14.53	[14.11; 14.98]			3765	[3723; 3808]	5.0	[5.0; 5.1]		
Bladder in women												
Guadeloupe	8	[6; 10]	1.4	[1.0; 2.0]	0.70	[0.52; 0.91]	3	[2; 4]	0.5	[0.3; 1.0]	0.50	[0.31; 0.76]
Martinique	6	[5; 9]	1.2	[0.9; 1.8]	0.56	[0.42; 0.74]	6	[5; 8]	1.1	[0.7; 1.7]	1.05	[0.77; 1.42]
French Guiana	2	[1; 3]	1.9	[0.8; 3.8]	0.73	[0.31; 1.43]	0	[0; 1]	0.2	[0.0; 1.0]	0.27	[0.03; 0.97]
Mainland France	2188	[2099; 2283]	2.3	[2.2; 2.4]			1178	[1154; 1202]	0.95	[0.92; 0.97]		
Kidney in men												
Guadeloupe	15	[12; 18]	5.2	[4.2; 6.4]	0.35	[0.29; 0.42]	5	[4; 7]	1.7	[1.2; 2.5]	0.34	[0.25; 0.46]
Martinique	12	[9; 14]	4.3	[3.5; 5.5]	0.26	[0.21; 0.32]	4	[3; 6]	1.4	[0.9; 2.2]	0.26	[0.17; 0.37]
French Guiana	6	[4; 9]	6.1	[4.1; 9.0]	0.46	[0.31; 0.65]	1	[1; 2]	1.5	[0.7; 2.8]	0.39	[0.19; 0.69]
Mainland France	7947	[7810; 8087]	14.8	[14.5; 15.0]			2934	[2896; 2972]	4.4	[4.3; 4.5]		
Kidney in women												
Guadeloupe	8	[6; 11]	2.5	[1.8; 3.4]	0.36	[0.27; 0.46]	3	[2; 5]	0.8	[0.5; 1.4]	0.46	[0.30; 0.67]
Martinique	8	[6; 10]	2.3	[1.7; 3.2]	0.34	[0.26; 0.44]	3	[2; 5]	0.6	[0.4; 1.3]	0.41	[0.26; 0.62]
French Guiana	4	[2; 6]	3.4	[2.0; 5.6]	0.57	[0.34; 0.91]	0	0 [0; 1]	0.6	[0.1; 1.6]	0.37	[0.10; 0.94]
Mainland France	4058	[3935; 4184]	6.3	[6.1; 6.5]			1446	[1420; 1473]	1.4	[1.42; 1.49]		

^a^Incidence mainland France: 2007–2016; Guadeloupe: 2008–2014; Martinique: 2007–2014; French Guiana: 2010–2014

^b^World-standardized rates: rates are standardized to the age structure of the world standard population and expressed per 100,000 person-years

^c^Ratios standardized to mainland France

In Guadeloupe and Martinique, the world-standardized incidence rates are among the highest in the world (respectively, 173.0 and 164.5 per 100,000 person-years) and are almost twice as high as the national average, estimated at 88.8 per 100,000 person-years (SIR for Guadeloupe: 1.91 [1.85–1.97] and for Martinique: 1.79 [1.73–1.84]) (Table 1).

In French Guiana, the world-standardized incidence rate is 94.4 per 100,000 person-years (Table 1).

In the French Overseas territories, although it has been declining for several years, mortality from prostate cancer remains more than twice as high as that observed in mainland France, at 23.0 per 100,000 person-years in Guadeloupe and Martinique, and 16.9 in French Guiana (vs 10.0 in mainland France) (Table 1). These rates place these 3 regions at the top of the list of regions of France with the highest mortality rates from prostate cancer.

### Bladder

There are 24 new cases of bladder cancer per year in Guadeloupe, 21 in Martinique and 7 in French Guiana, with a male-to-female ratio of 2. In men, the highest world-standardized incidence rate is observed in French Guiana, at 5.9 per 100,000 person-years. The rates in Guadeloupe and Martinique are, respectively, 4.9 and 4.1 per 100,000 person-years. In women, the differences are less marked, with a world-standardized incidence rate of 1.9 in French Guiana, 1.4 in Guadeloupe and 1.3 in Martinique (Table 1). These rates place the French Overseas territories among the regions with the lowest incidence of bladder cancer. The under-incidence is more pronounced in men (56–72%) than in women (27–44%) (Table 1).

In the French Overseas territories, the world-standardized mortality rate from bladder cancer varies from 1.5 in French Guiana to 1.8 in Guadeloupe and 2.0 in Martinique in men. In women, it ranges from 0.2 in French Guiana to 0.5 in Guadeloupe and 1.1 in Martinique (Table 1).

### Kidneys

Kidney cancer accounts for around 20 new cases per year, respectively, in Guadeloupe and Martinique, and 10 in French Guiana. World-standardized incidence rates are slightly higher in French Guiana than in Guadeloupe and Martinique, but the incidence of kidney cancer remains nonetheless lower in these 3 regions than in mainland France. World-standardized incidence rates in men are 4.3 per 100,000 person-years in Martinique, 5.2 in Guadeloupe and 6.1 in French Guiana, and 2.3, 2.5 and 3.4, respectively, in women (Table 1).

Trends in kidney cancer mortality largely follow those of incidence in the French Overseas territories, with world-standardized mortality rates in men of 1.7 per 100,000 person-years in Guadeloupe, 1.4 in Martinique, and 1.5 in French Guiana, while in women, corresponding rates were 0.8 in Guadeloupe and Martinique and 0.6 in French Guiana. All these rates are lower than those observed in mainland France (Table 1).

## Discussion

The use of prostate-specific antigen (PSA) assays as a screening tool for prostate cancer has participated to the increase in the incidence of prostate cancer over the last decade. Mortality has been declining slowly but surely since the 1990s [19]. Prostate cancer was responsible for slightly more than 8,700 deaths per year in mainland France from 2007 to 2014, accounting for 9.9% of cancer-related deaths in men. This low proportion is explained by the excellent prognosis of prostate tumors diagnosed at an early stage. For cases diagnosed recently (2005–2010), net survival at 5 years was 94% [20].

The only established risk factors for prostate cancer are individual, namely age, ethnic origin, and a family history of prostate cancer. Among the environmental risk factors, endocrine disruptors are considered as a potential risk factor for several cancers, including prostate cancer, due to their ability to disrupt the hormonal system. Among these, pesticides, notably chlordecone (classed as possibly carcinogenic by the IARC), are associated with an increased risk of prostate cancer. However, for pesticides, as well for diet, which is also suspected to play a role in the genesis of prostate cancer, existing data warrant consolidation [21, 22].

In Guadeloupe, incidence of prostate cancer increased between 2008 and 2012, but has been declining since 2013. In parallel, there has been a decrease in mortality. Net survival at 5 years was 91% for the period 2008–2012 [8]. In Martinique, net survival of 98% was reported in the Concord 2 study [23]. In both these regions, contamination of the ground and water by chlordecone has been associated with the high observed incidence of prostate cancer [11]. Additional studies are ongoing to identify genetic susceptibilities that may be related to exposure to this endocrine disruptor.

The incidence observed in Guadeloupe and Martinique is also higher than rates elsewhere in the Caribbean in countries with a cancer registry, namely: 123.1 for Barbados, 123.9 for Trinidad & Tobago in 2012 [24], and also higher than the rate of 139 per 100,000 person-years observed in the African-American population based on data from the SEER programme for the period 2011–2015 [25].

In French Guiana, the incidence rate is not significantly different to the rate observed in mainland France (SIR 1.04 [0.94–1.15]).

Our study provides the first cancer incidence and mortality data for all the French overseas territories in the Caribbean, and could be of use to patients, the scientific community as well as health authorities and decision-makers. The presentation of these data for the three regions of Martinique, Guadeloupe and French Guiana makes it possible to highlight similarities and differences in incidence and mortality between regions for the main urological cancers.

The incidence of bladder cancer is highest in developed countries with a strong male predominance (four men for one woman affected) [26]. In the French overseas territories, incidence is considerably lower than in mainland France, where the national estimate is 14.5 per 100,000 person-years in men and 2.3 in women. In mainland France, 4.8% of incident cancer cases occurred in men and 1.4% in women. In men, incidence has been declining steadily since the 1990s, whereas it has been increasing slightly in women since 2005 [19]. However, these figures should be interpreted with caution due to the high variability in recording practices and in coding between registries over time [26]. Mortality has been decreasing consistently since the 1990s [19]. Bladder cancer was responsible for 4.2% of cancer-related deaths in men, and 1.9% in women. Net survival at 5 years for bladder cancers diagnosed between 2005 and 2010 was 50% for men and 43% for women [27].

Renal cancer has high incidence in North American and Europe [24, 28]. Around 60% of new cases occur in men [29]. France counts among the Western European countries with the highest incidence rates [28]. Kidney cancer accounted for 4% of all incident cancer cases in men and 2.6% in women in mainland France between 2007 and 2016. There is also a clear male predominance in France, with an average 7947 new cases in men annually, compared to 4058 in women, over the period 2007–2016. The incidence of kidney cancer has been increasing steadily since 1980, in both men and women, and this phenomenon appears to be at least partially linked to the increased frequency of imaging exams performed for other indications, which facilitates fortuitous discovery of early-stage kidney cancers.

A study assessed incidence and mortality rates of upper tract urothelial cancers by analyzing Surveillance, Epidemiology and End Results registry for the 2004–2016 period. This study showed that overall incidence rates decreased significantly from 1.3 per 100,000 person-years to 1.1 during this period for both renal pelvis and ureteral cancers. Unfortunately, according to stage at diagnosis, metastatic patients had increased incidence rates in this study [30]. Conversely, mortality from renal cancer remains stable [19], with an average of 2934 deaths per year in men, and 1446 deaths per year in women in mainland France between 2007 and 2014, reflecting the increased survival of patients with renal cancer. For renal cancers diagnosed between 2005 and 2010, net survival at 5 years was 71% in both sexes [27]. Obesity, tobacco smoking and arterial hypertension are the main risk factors identified [31].

## Conclusion

Identifying the profile of patients with urological cancers in the French overseas territories is key to understanding the needs of patients in these regions, and consequently, for informing public health policy regarding management and therapy. Providing reliable and up-to-date epidemiological indicators relating to cancer incidence and mortality from the Caribbean will help to identify risk factors specific to this geographical area, with a view to developing multicentre studies to investigate environmental and socio-cultural factors that may explain the observed cancer trends. Specifically, in the framework of the public health chlordecone campaign, there is a compelling need to identify the factors that explain prostate cancer, and the results of the present study may serve as a basis for further investigations into the causal relationship between pesticide exposure and prostate cancer.

## Data Availability

Not applicable.

## Abbreviations

RR: Relative risk
SIR: Standardized incidence ratio
SMR: Standardized mortality ratio
WHO: World Health Organization
